# Developing a device for simultaneously investigating pivoting neuromuscular control and muscle properties toward a multi-axis rehabilitation

**DOI:** 10.1371/journal.pone.0304665

**Published:** 2024-07-08

**Authors:** Song Joo Lee, Hyunah Kang, Keun-Tae Kim, Sang Hoon Kang

**Affiliations:** 1 Bionics Research Center, Korea Institute of Science and Technology (KIST), Seoul, Republic of Korea; 2 Division of Bio-Medical Science & Technology, KIST School, Korea University of Science and Technology, Seoul, Republic of Korea; 3 Department of Mechanical Engineering, Ulsan National Institute of Science and Technology (UNIST), Ulsan, Republic of Korea; 4 Department of Physical Therapy and Rehabilitation Science, University of Maryland, Baltimore, Maryland, United States of America; Brunel University London, UNITED KINGDOM

## Abstract

Understanding the pivoting neuromuscular control of the lower limb and its associated muscle properties is critical for developing diagnostic and rehabilitation tools. However, to the best of our knowledge, a device that can evaluate these factors simultaneously remains lacking. To address this gap, a device that can investigate pivoting neuromuscular control and associated muscle properties was developed in this study. The proposed device consisted of a pivoting mechanism and height-adjustable chair with a brace interface. The device can control a footplate at various speeds to facilitate pivoting stretching and quantify neuromuscular control. Time-synchronized ultrasonographic images can be acquired simultaneously to quantify muscle properties during both active and passive pivoting movements. The muscle displacement, fascicle length/displacement, pennation angle, pivoting stiffness, and pivoting instability were investigated using the proposed device. Further, the feasibility of the device was demonstrated through a cross-sectional study with healthy subjects. The proposed device successfully quantified changes in muscle displacement during passive and active pivoting movements, pivoting stiffness during passive movements, and neuromuscular control during active movements. Therefore, the proposed device is expected to be used as a research and therapeutic tool for improving pivoting neuromuscular control and muscle functions and investigating the underlying mechanisms associated between muscle properties and joint movement in the transverse plane.

## Introduction

Major movements in the ankle and knee joint occur in flexion/extension. However, joint instability attributed to neurological disorders and lower limb injuries occur in the internal/external rotation and/or the abduction/adduction. Especially in-toeing or out-toeing gait patterns (in the transverse plane) can often be observed among individuals with a neurological disorder such as cerebral palsy [1]. Moreover, individuals with sport-related injuries (e.g., anterior cruciate ligament injuries) show joint instability in the transverse plane [2]. With this in mind, a pivoting off-axis elliptical trainer system was developed for individuals with musculoskeletal injuries and neurological disorders [3]. This device provided challenging pivoting tasks in the transverse plane while rehabilitation exercise (stepping) on the trainer, termed pivoting off-axis intensity adjustable neuromuscular control training [4], and can be used for pre- and post-neuromuscular evaluations with the training [1, 4–7]. However, muscle properties have not been investigated using this device although subjects pivoted in the transverse plane in its current form.

Studies that use devices for investigating muscle properties during movements and before and after stretching tasks are scarce and have investigated neuromuscular control and muscle properties in the sagittal plane during passive stretching or active movement [1, 3, 4, 6, 8, 9]. Given the importance of pivoting neuromuscular control in preventing pivoting-related injuries and reducing in-toeing gait [1, 4–6], it would be beneficial to concurrently investigate neuromuscular control and muscle properties during active and passive movements in the transverse plane for both clinicians and patients. A multi-level concurrent investigation of neuromuscular control and muscles could provide a better understanding of the underlying mechanism of diseases/injuries and changes followed by promising training. Therefore, there is a strong need for a device that can investigate the neuromuscular control and related muscles during the pivoting movement in the transverse plane. However, to the best of the authors’ knowledge, no such device is available yet.

Therefore, the objective of this study was to develop a prototype device to concurrently investigate neuromuscular control and muscle properties in the transverse plane with the long-term goal of complementing the clinical assessment. The feasibility of the initial prototype of the proposed device for lower limb multi-level assessment was demonstrated through experiments with healthy subjects. It was hypothesized that the prototype device could quantify changes in muscle displacement during passive and active pivoting movements, pivoting stiffness during passive movements, and neuromuscular control during active movements.

## Materials and methods

### Device for Simultaneously Investigating Pivoting Neuromuscular Control and Muscle Properties (DIPNM)

**Fig 1:**
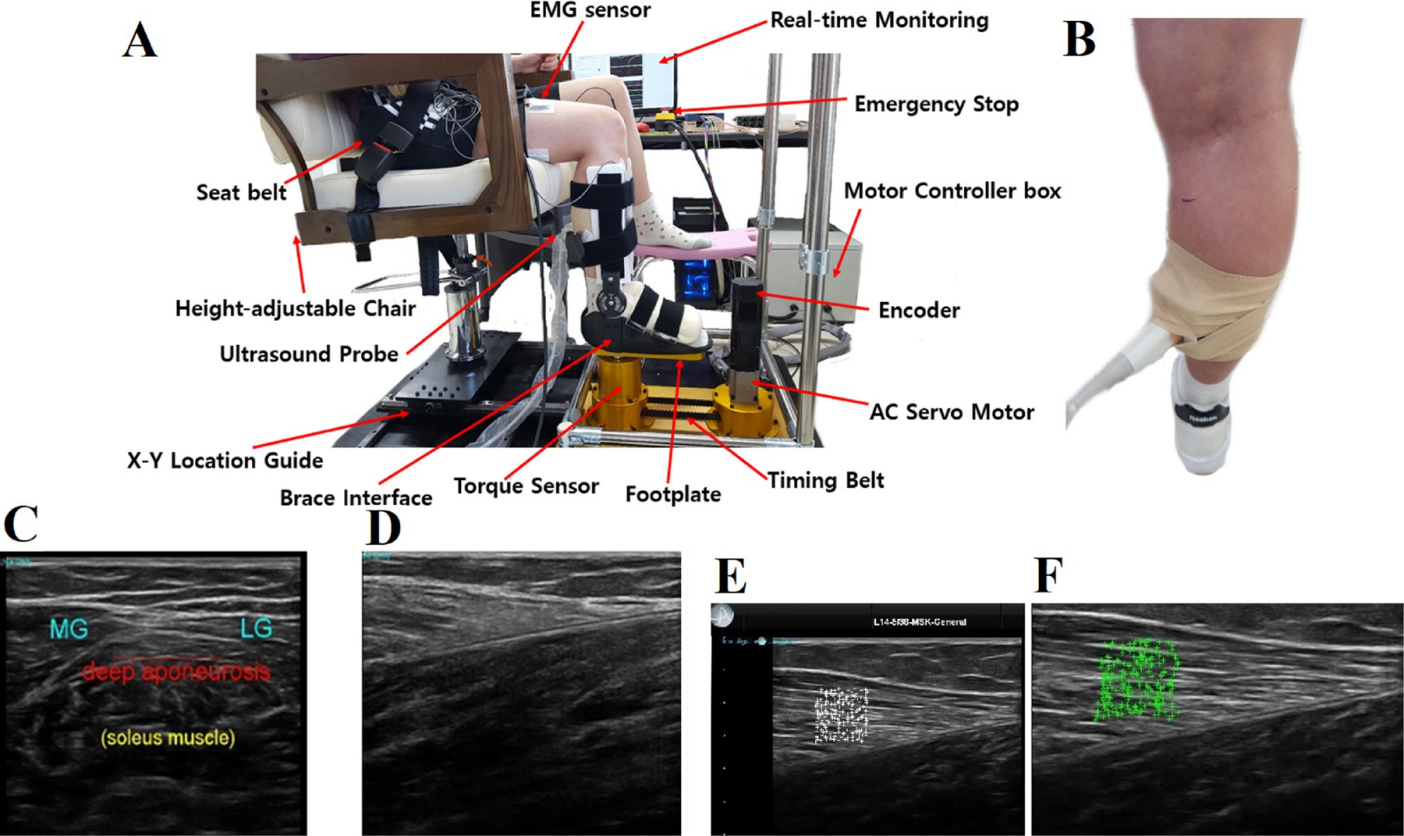
Developed device for simultaneously investigating pivoting neuromuscular control and muscle properties (DIPNM). (A) DIPNM consisting of a pivoting control mechanism, height-adjustable chair module, and brace interface. (B) Ultrasound probe attached to the target muscle via a Styrofoam mold wrapped by a cohesive bandage around the medial gastrocnemius muscle. (C) Finding the medial gastrocnemius (MG) and lateral gastrocnemius (LG) ultrasonographic image in the transverse plane. (D) Medial gastrocnemius ultrasonographic image. (E),(F) Muscle fascicle displacement tracking during stretching of the medial gastrocnemius. The left (E) white cross marks indicate the identified muscle fascicle within the 8 mm × 8 mm box at the initial position. The right (F) green cross marks indicate tracked cross marks at each frame to compute the displacement from the initial position.

A device for simultaneously investigating pivoting neuromuscular control and muscle properties (DIPNM) was developed (Fig 1A). The DIPNM comprised a pivoting control mechanism, height-adjustable chair module with a hydraulic cylinder, and brace interface for an ultrasound probe to investigate muscle properties.

The pivoting control mechanism was operated by a custom-made LabVIEW program that commanded a torque signal to a motor driver (XDL-L7SA004B, LS Mecapion, South Korea) that regulated a 400 W alternating current servomotor (XGT Servo, LS Mecapion, South Korea). This motor had a maximum speed of 3000 revolutions per minute (RPM), 15:1 speed reducer, and built-in encoder (Resolution: 3000 pulse/revolution) that measured the pivoting angle. The torque was then transmitted to the footplate via a timing belt connecting the output side of the speed reducer and footplate. The interaction torque exerted on the foot in the pivoting direction was measured by a torque sensor (500 in-lb, Fultek, USA) positioned right beneath the footplate.

The height-adjustable chair module used in this study comprised a base, hydraulic cylinder, and chair equipped with seat belts (Fig 1A). The base of the chair incorporated *x*-*y* linear guides that allowed for adjustment of anteroposterior and mediolateral positioning to align the tibial rotation axis (the long axis projected to the footplate near the heel [3]) with the footplate rotation axis, thereby accommodating variations in leg length and size. The hydraulic cylinder facilitated chair height adjustment to ensure a consistent knee flexion angle for participants of varying heights. Seat and thigh straps were used to control trunk and thigh motion effectively during both passive and active tasks.

The brace interface comprised a brace attached to the top of the footplate with straps (Fig 1A), which allowed an ultrasound probe to be attached to the target lower limb muscle (e.g., medial gastrocnemius (MG) muscle; Fig 1B) without physical interference. The brace interface provided adequate support for the foot to be well-secured during the pivoting motion so that the pivoting torque and motion could be measured reliably. The pivoting torque and angle measured by DIPNM were time-synchronized with ultrasonographic images and electromyographic (EMG) activities using a handheld switch.

The device was designed to allow the controlled movement of the footplate and foot strapped to it within predetermined pivoting angle and torque limits. Before the start of each trial, the pivoting range of motion (ROM) was measured, and from this, the limits for internal/external pivoting angle and internal/external pivoting torque were determined for each subject. Then, the footplate moved a foot at a selected slow speed (e.g., 2°/s) while remaining within the predetermined limits. DIPNM immediately stopped and resumed movement in the opposite direction when the angle limit or the torque limit was reached in one direction of movement, thereby maintaining the same slow constant speed. These safety limits ensured the safety of the subjects and provided consistency across subjects during the pivoting stretching and active pivoting neuromuscular tasks described in the following.

Slow movements within predetermined limits were utilized for two different tasks. The first task involved the passive stretching of a subject’s lower limb in pivoting directions while the subject was relaxing with no muscular contraction of the lower limb. The slow constant speed stretching allowed us to quantify passive pivoting stiffness by measuring the pivoting angle and torque while simultaneously tracking the movement of the muscle fascicles. The second task was an active pivoting neuromuscular task wherein subjects were instructed to maintain a neutral position by resisting the slow constant speed back-and-forth movement of DIPNM within the predetermined angle and torque limits. The pivoting neuromuscular control performance of subjects could be investigated by comparing the standard deviation of the pivoting angle, termed pivoting instability.

Muscles could be monitored for both passive stretching and active pivoting neuromuscular tasks. The ultrasonographic video was acquired in a time-synchronized manner using a handheld switch, allowing the simultaneous examination of the ultrasonographic image, torque, and angle signals. The proposed device uses a Styrofoam mold to wrap the ultrasound probe around the lower leg with a cohesive bandage, ensuring consistent probe position and compression pressure throughout the tasks. Thus, the position and compression pressure of the ultrasound probe could be consistent throughout the tasks, and the quality of the ultrasonographic images could be more uniform compared to holding the probe by hand (Fig 1B).

The pivoting torque and angle were acquired using the custom LabVIEW software at a sampling rate of 1,000 Hz. The ultrasonographic image video was acquired using the L14-5 linear probe of the sonixTOUCH system (Ultrasonix, Canada) with an acquisition rate of 7.76 Hz and a frame rate of 25 Hz.

### Safety considerations

For safety, subjects were provided with a mechanical switch that allowed them to stop DIPNM at their discretion. In addition, the operator was able to activate a software-based stop button and deactivate another enable switch when necessary. Further, real-time monitoring was used to ensure that the pivoting torque and position remained within the predetermined limits.

### Subjects

Seven healthy subjects, community-dwelling adults conveniently sampled from the population of South Korea, were enrolled in the study in 2016 to evaluate the feasibility of DIPNM (Table 1). As the objective of this study was to evaluate the feasibility of the initial prototype of DIPNM, based on our previous device development study related to pivoting training [3] and a pilot study on quantifying muscle properties, a sample size of seven was deemed sufficient to provide a power of 0.8, effect size (d) of 1.28, and alpha (α) level of 0.05. Before the experiment, all subjects read and signed an informed consent form approved by the Institutional Review Board of the Korea Institute of Science and Technology (2016–014). Participant recruitment and data collection were performed at KIST under a research setting in December 2016.

**Table 1:** Subject’s characteristics (N = 7).

Parameter	Mean (standard deviation)
Age (year)	37.7 (17.2)
Sex	5F/2M
Body Mass (kg)	64.4 (15.0)
Height (m)	1.65 (0.08)
Thigh circumference (cm)	43.5 (3.0)
Calf circumference (cm)	36.8 (4.1)
Lower leg length (cm)	39.1 (3.9)
Higher leg length (cm)	38.0 (4.3)

### Protocol for the simultaneous investigation of pivoting neuromuscular control and muscle properties

As an illustration of the potential clinical application of DIPNM, the effects of pivoting stretching on pivoting neuromuscular control, muscle fascicle length, and pennation angle were investigated using DIPNM. The following protocol was designed to quantify muscle fascicle displacement and pivoting stiffness during pivoting stretching and muscle fascicle length, pennation angle, and pivoting neuromuscular control in terms of pivoting instability before and after pivoting stretching.

**Fig 2:**
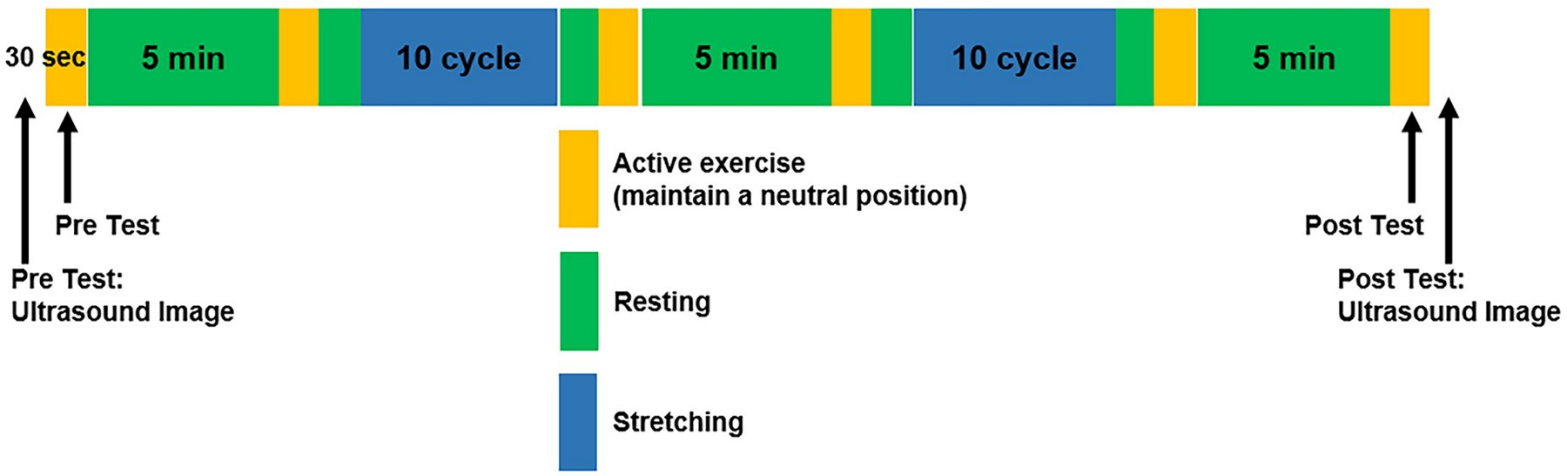
Experimental protocol using the DIPNM. Pre-I test at the very beginning of the protocol (left-most, Pre test: Ultrasound image) and Post-I test at the very end of the protocol (right-most, Post test: Ultrasound image) are tests measuring the fascicle length and pennation angle of MG while the subjects are standing. Active exercise (yellow box) indicates active pivoting neuromuscular task, and stretching represents passive stretching.

The ultrasonographic images of the MG were obtained before and after the subjects sat on the chair of DIPNM while standing on the floor (at both ends of the protocol as Pre-I and Post-I tests, respectively; Fig 2). Each subject was instructed to place their feet shoulder-width apart and stand on their toes. After aligning the deep aponeurosis of the MG muscle parallel to the bottom of the ultrasonographic image (Fig 1C and 1D) [1], the ultrasonographic images of MG were obtained by positioning the ultrasound probe placed in a Styrofoam mold on the belly of MG. The insertion point of the MG from the Achilles tendon was identified (Fig 1C and 1D), and the ultrasound probe was secured in place with cohesive bandages. This allowed the probe to remain attached to the skin without changing its position or compression pressure (Fig 1B). Then, each subject sat on the chair at an ankle dorsiflexion angle of 90°, knee flexion angle of 90°, and hip flexion angle of 85° with the ultrasound probe attached (Fig 1B). The ultrasonographic image video was acquired during all active and passive stretching tasks except the rest periods to quantify the maximum displacement of the MG muscle fascicle. The pivoting torque and angle were monitored and measured throughout the tasks in real-time with the specific tasks mentioned below.

Each subject performed a series of stretching and active pivoting neuromuscular tasks (Fig 2) to examine the effects of pivoting stretching on pivoting neuromuscular control and muscle properties. Subjects performed six 30-s active pivoting neuromuscular tasks (yellow intervals) with instructions to maintain the footplate in a neutral position. Data from the Pre-test (first yellow interval), Stretching 1 (first blue interval), Stretching 2 (second blue interval), and Post-test (last yellow interval) were analyzed for this study.

During the stretching task, the subject’s leg in the pivoting direction was stretched for ten cycles (~ 4 min each; Stretching 1 and Stretching 2 of blue intervals in Fig 2) while the subject relaxed with no muscle activation. Rest periods (green intervals) were provided to prevent fatigue (Fig 2). For each task, the DIPNM footplate had a fixed limit of ± 10° for the pivoting angle and a limit of ± 8 Nm for the pivoting torque. The DIPNM footplate was operated at a constant speed of 2°/s. Therefore, if the device reached the predetermined limit for either the pivoting angle or the pivoting torque in a given direction, DIPNM would not provide additional stretching in that direction, thereby ensuring consistency and safety.

### Data analysis

#### Concurrent ultrasonographic quantification of muscle fascicle displacement during pivoting stretching

**Fig 3:**
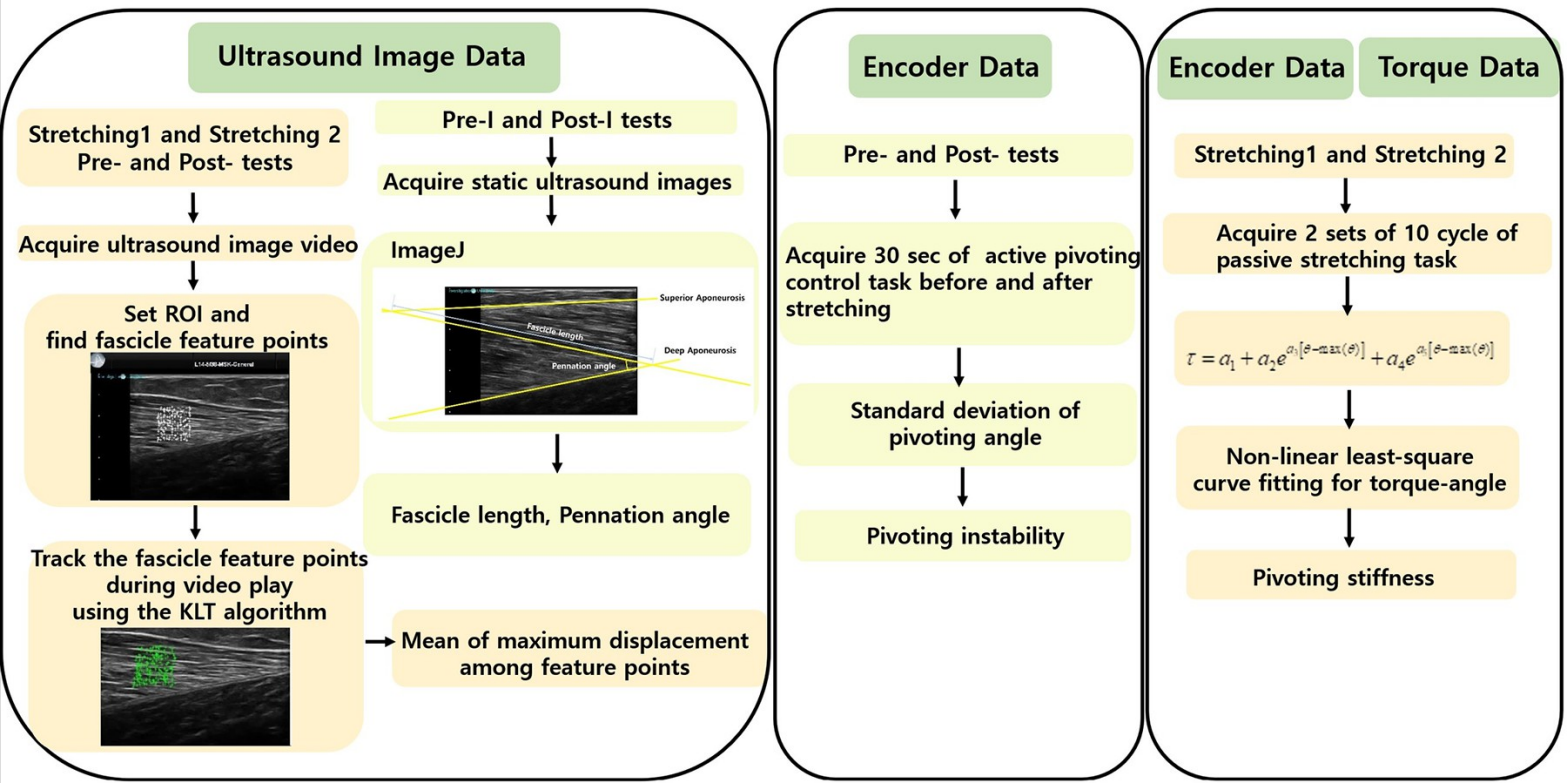
Flowchart for analyzing the ultrasonographic image, encoder, and torque data for each test.

Changes in the fascicle length (i.e., muscle fascicle displacement) were determined by analyzing the collected brightness mode (B-mode) ultrasonographic image video during Pre-test, Post-test, Stretching 1, and Stretching 2. Feature points within an 8 mm by 8 mm square box of the fascicle image (Fig 1E and 1F) were identified based on the minimum eigenvalue algorithm [10, 11] among potential feature extraction methods [12–14] to obtain the muscle fascicle displacement from the ultrasonographic image video, and their displacement was tracked by monitoring their positions throughout the frames. The Kanade–Lucas–Tomasi (KLT) feature tracking algorithm, executed in MATLAB (Mathworks, Ntick, MA, USA), was used to track the trajectory of each point. The maximum displacement was recorded for each feature point indicated as each cross point in Fig 1E and 1F, and the mean of the maximum displacement among feature points was obtained and termed the mean value of the maximum displacement of the muscle fascicle. The muscle fascicle length and pennation angle of the MG were assessed using ImageJ software (National Institutes of Health, Bethesda, MD, USA). This assessment was conducted based on the ultrasonographic images obtained during the Pre-I and Post-I tests (Fig 2), with each subject positioned in a standing posture on the ground. The muscle fascicle length was determined by measuring the distance along the fascicular path from the deep to the superficial aponeuroses [15] (Fig 3). The pennation angle was quantified as the positive angle formed between the fascicle line and the deep aponeurosis [16, 17] (Fig 3).

#### Pivoting neuromuscular control and stiffness

Pivoting neuromuscular control was defined in terms of pivoting instability, which represents the ability to control the pivoting direction movement during the active pivoting neuromuscular control task. Pivoting instability was quantified as the standard deviation of the pivoting angle [4] during the active pivoting neuromuscular task, namely Pre-test and Post-test. Pivoting stiffness was estimated by fitting the measured pivoting angle and torque curves to [18, 19]

$$\tau =a_{1}+a_{2}e^{a_{3}[\theta -\max(\theta )]}+a_{4}e^{a_{5}[\theta -\max(\theta )]},$$ (1)

where *τ* and *θ* represent pivoting torque and pivoting angle, respectively; *a*_*k*_s (*k* = 1, 2, …, 5) represent constants to be determined for fitting the torque-angle curve. After *a*_*k*_s were determined by the nonlinear least-squares curve fitting, stiffness was determined by taking the derivative of the right side of (1) with respect to *θ* [18]. The average stiffness at the 0° pivoting external angle across cycles was obtained for each subject. Pivoting stiffness was estimated during the first (Stretching 1) and second (Stretching 2) stretching periods. A flowchart demonstrating the aforementioned analysis for each specific test is provided in Fig 3.

### Statistics

We used the Wilcoxon signed-rank test to assess the statistical significance of differences in muscle properties (MG muscle fascicle length and pennation angle) between the Pre-I and Post-I tests, pivoting instability between the Pre- and Post-tests, and pivoting stiffness between Stretching 1 and Stretching 2. Further, we used the Wilcoxon signed-rank test to evaluate the mean maximum displacement of the MG muscle fascicle between Stretching 1 and Stretching 2, as well as between the Pre-test and the Post-test. The significance level was set at 0.05.

## Results

All seven subjects participated in the study (Table 1).

### Muscle properties before and after

**Fig 4:**
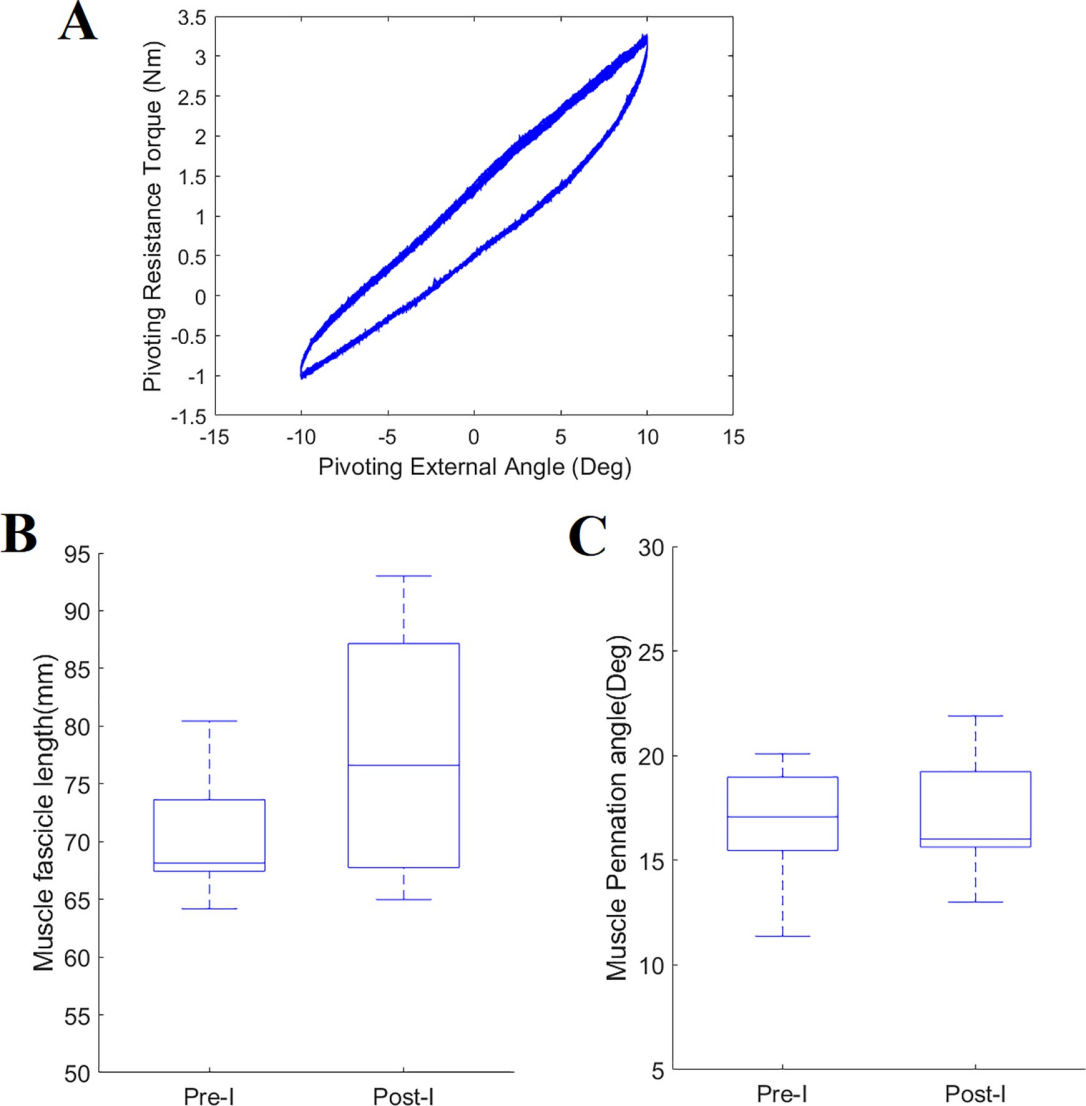
Passive stretching and muscle properties before and after. (A) Pivoting angle (Positive: external pivoting) and resistance torque (Positive: internal pivoting) relationship (hysteresis loop) during multiple pivoting stretching cycles. (B) MG muscle fascicle length and pennation angle at pre- and post-I tests. (C) MG muscle pennation angle at the pre-I and post-I test. Each box indicates the 25^*th*^ (Q1) and 75^*th*^ (Q3) percentiles of the data. A line across and within each box indicates median (50^*th*^ (Q2) percentile). The whisker indicates the highest and lowest values of the data that did not deviate greater than 1.5 times the interquartile range (IQR) from the limits of a box. The grey open circle indicates an outlier that deviated more than 1.5 times the IQR from the box limits.

DIPNM safely stretched the lower limb in the pivoting direction while simultaneously measuring muscle fascicle displacement and pivoting angle and torque for the Stretching 1 and Stretching 2 tasks (Fig 4A).

The initial MG fascicle length (Median (IQR: 25^th^, 75^th^ percentile): 68.14 (67.44, 73.63) cm) before the Pre-test was not significantly different from the final measurement length (76.62 (67.76, 87.15) cm) after the Post-test (Fig 4B). The initial MG pennation angle (17.08 (15.47, 18.97°) before the Pre-test was not significantly different from the final measurement angle (16.02 (15.63, 19.23°; Fig 4C) after the Post-test.

### Muscle characteristics during the passive stretching and active pivoting neuromuscular tasks

**Fig 5:**
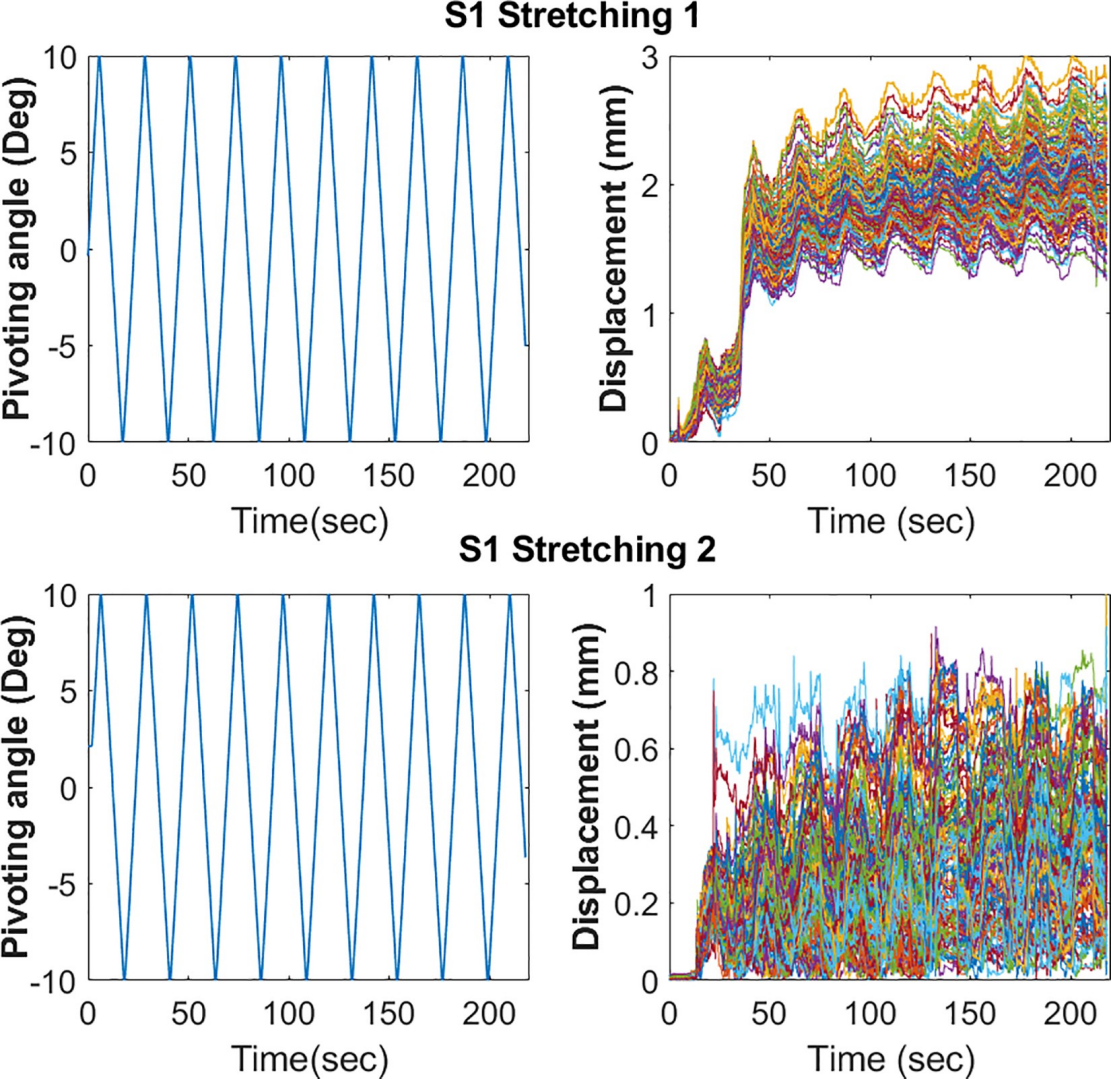
Representative cases of muscle fascicle displacement during internal/external pivoting stretching. Each color graph indicates each identified muscle fascicle displacement.

Muscle fascicle displacement was investigated during the pivoting stretching period. A representative case is shown in Fig 5.

**Fig 6:**
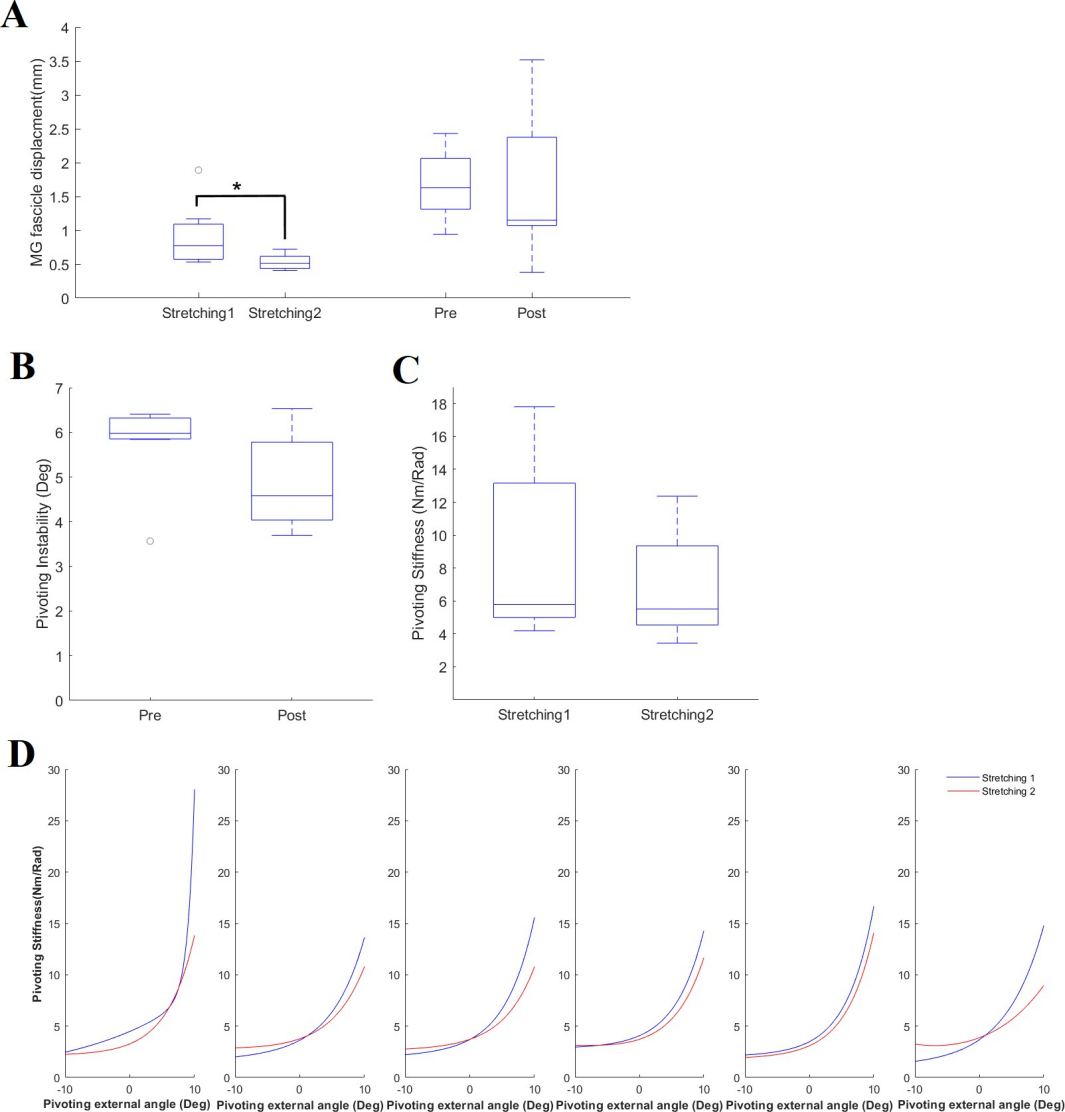
Muscle characteristics. (A) Boxplots of the MG mean value of maximum muscle fascicle displacement during the Stretching 1 and Stretching 2 tasks and the Pre- and Post-tests. (B) Pivoting instability at the Pre- and Post-tests. (C) Mean pivoting stiffness. Each box indicates the 25^th^ (Q1) and 75^th^ (Q3) percentiles of the data. A line across and within each box indicates the median (50^th^ (Q2) percentile). The whisker indicates the highest and lowest values of data that do not deviate greater than 1.5 times the interquartile range (IQR) from the limits of a box. The grey open circle indicates an outlier that deviated more than 1.5 times the IQR from the box limits. (D) Representative pivoting stiffness from six consecutive individual hysteresis cycles at Stretching 1 (blue graph) and Stretching 2 (red graph). The six cycles were arranged by time, starting at the leftmost.

A significant reduction in the mean value of the maximum displacement of the muscle fascicle was found from Stretching 2 (0.51 (0.44, 0.62) mm) compared to Stretching 1 (0.78 (0.57, 1.09) mm; p = 0.016; Fig 6A). During the active pivoting neuromuscular task, the mean value of the maximum displacement of muscle fascicle from the Pre-test (1.63 (1.31, 2.07) mm) was not significantly different from that from the Post-test (1.15 (1.07, 2.38) mm).

### Effects of pivoting stretching on pivoting neuromuscular control and stiffness

A decreasing trend was observed for pivoting instability: from 5.98 (5.85, 6.32° in the Pre-test to 4.58 (4.04, 5.78° in the Post-test (Fig 6B). Compared to Stretching 1, there was a trend of a slight decrease in the mean pivoting stiffness found in Stretching 2 (Fig 6C). When considering the individual hysteresis loop of the torque-angle graph, there was a tendency for the pivoting stiffness to decrease as the number of stretching cycles increased (Fig 6D).

## Discussions

To address the urgent clinical and scientific needs, the DIPNM was developed to investigate pivoting neuromuscular control and muscle characteristics simultaneously during passive pivoting stretching and active pivoting neuromuscular control. Although previous studies developed a device to study pivoting neuromuscular control during functional stepping [3–7], DIPNM is the first device to study not only pivoting neuromuscular control but also muscle characteristics such as muscle fascicle length, displacement, and pennation angle. Our feasibility study in healthy subjects demonstrated that muscle fascicle displacement can be monitored during pivoting passive and active motion in DIPNM.

Our results from testing healthy subjects showed that the pennation angle and muscle fascicle length did not change significantly after performing the tasks on DIPNM. In this study, the pennation angle of the MG before the Pre-test (17.08 (15.47, 18.97°) was comparable to a previous report of 17.1 ± 2.4° at the full knee extension and 0° ankle dorsiflexion [8]. Therefore, our method of quantifying the pennation angle and muscle fascicle length is acceptable.

There was a trend toward a reduction in pivoting instability after stretching. Previous studies of ankle stretching in the sagittal plane in stroke survivors and children with cerebral palsy suggested that stretching may help to improve ankle control, reduce joint stiffness, and increase passive ROM in the sagittal plane [8, 9]. Similarly, the two stretching tasks (Stretching 1 and Stretching 2) in our study might help control the footplate in the neutral position during the active pivoting neuromuscular control task.

This is the first study that was able to evaluate passive pivoting stiffness and muscle displacement simultaneously. The short 20 cycles could not show the difference in passive pivoting stiffness in healthy individuals; however, as shown in Fig 6D, an individual cycle-based pivoting stiffness can be quantified in terms of the pivoting angle. Our approach and method can be further used to investigate the effects of stretching on passive pivoting stiffness and pivoting neuromuscular control.

It is interesting to observe muscle thixotropy with the developed device (Fig 5). Muscle thixotropy indicates that the stiffness of the muscle is reduced following active or passive movement [20]. In Fig 5, the representative case shows the drastic change in muscle displacement in the first 50 s (~ two stretching cycles), suggesting muscle thixotropy due to stretching. In Fig 6D, there is also a representative reduction in the pivoting stiffness between the first and last stretching cycles. This phenomenon was not observed during the second stretching period, which could be related to the time-dependent behavior of the muscle because of previous stretching [20]. Previous studies showed that muscle thixotropy influence proprioception and postural control [20]. Further studies are required, especially in patients with neurological disorders, to understand the muscle thixotropy behavior of pivoting stretching and neuromuscular performance.

Muscle fascicle length and pennation angle did not change in our healthy subjects after 20 pivoting cycles. It means that our stretching in the pivoting direction did not cause any yielding and performed safely. It would be interesting to investigate whether muscle architecture, such as fascicle length and pennation angle, could be modulated by long-term stretching in subjects with pathological conditions.

Using DIPNM, we demonstrated that the pivoting neuromuscular performance and pivoting stiffness can be characterized together with muscle fascicle displacement during the tasks. Moreover, muscle fascicle length and pennation angle can be investigated; such an approach can further help develop a diagnostic tool and find an underlying mechanism of how pivoting neuromuscular control, pivoting stiffness, and muscle displacement can be associated. Recording torque and angle at 1,000 Hz via DIPNM allows for additional, synchronized EMG data measurement, enriching the dataset and potentially facilitating the long-term goal of complementing clinical assessments.

The study is limited to reporting the feasibility of the device prototype (i.e., DIPNM) to quantify pivoting neuromuscular control and muscle properties simultaneously only among seven healthy subjects, as it is a development study. An immediate future study will evaluate the feasibility of this DIPNM with a larger sample size and with individuals with a sports-related injury or a neurological disorder to strengthen the validity of the study results.

## Conclusion

This study developed and demonstrated the feasibility of DIPNM, the first device to investigate muscle properties and pivoting neuromuscular control during passive and active pivoting movements. Further studies in subjects with deficits in pivoting neuromuscular control and individuals with neurological disorders are expected to help further develop a diagnostic and rehabilitation tool to improve pivoting neuromuscular control and overall lower extremity function.

## Supporting information

S1 Data The data used to build the graphs (Figs 4, 5 and 6). (MAT)
